# Imaging the Galápagos mantle plume with an unconventional application of floating seismometers

**DOI:** 10.1038/s41598-018-36835-w

**Published:** 2019-02-04

**Authors:** Guust Nolet, Yann Hello, Suzan van der Lee, Sébastien Bonnieux, Mario C. Ruiz, Nelson A. Pazmino, Anne Deschamps, Marc M. Regnier, Yvonne Font, Yongshun J. Chen, Frederik J. Simons

**Affiliations:** 1 Université Côte d’Azur/CNRS/OCA/IRD, Géoazur, Sophia Antipolis 06560 France; 2 Department of Geosciences, Princeton University, Princeton, NJ 08540 USA; 3 School of Ocean Science and Engineering, SUSTech, 518055 Shenzhen, China; 4 Department of Earth and Planetary Sciences, Northwestern University, Evanston, IL60208 USA; 5 Instituto Geofísico, Escuela Politécnica Nacional, 2759 Quito, Ecuador; 6 INOCAR, 5940 Guayaquil, Ecuador; 7 Present Address: Université Côte d’Azur/CNRS, Laboratoire I3S, 06560 Sophia Antipolis, France

## Abstract

We launched an array of nine freely floating submarine seismometers near the Galápagos islands, which remained operational for about two years. P and PKP waves from regional and teleseismic earthquakes were observed for a range of magnitudes. The signal-to-noise ratio is strongly influenced by the weather conditions and this determines the lowest magnitudes that can be observed. Waves from deep earthquakes are easier to pick, but the S/N ratio can be enhanced through filtering and the data cover earthquakes from all depths. We measured 580 arrival times for different raypaths. We show that even such a limited number of data gives a significant increase in resolution for the oceanic upper mantle. This is the first time an array of floating seismometers is used in seismic tomography to improve the resolution significantly where otherwise no seismic information is available. We show that the Galápagos Archipelago is underlain by a deep (about 1900 km) 200–300 km wide plume of high temperature, with a heat flux very much larger than predicted from its swell bathymetry. The decrease of the plume temperature anomaly towards the surface indicates that the Earth’s mantle has a subadiabatic temperature gradient.

## Introduction

A global coverage of seismic stations has been – and still is – essential for the estimation of seismic hazard, the monitoring of nuclear explosions, and for developing an understanding of the internal structure of the Earth and the tectonics of plates at its surface. With the advent of seismic tomography, slight variations observed in the travel times of seismic signals have allowed us to map temperature and compositional anomalies at depth. Ever since the earliest seismograph networks were established more than a century ago, the number and quality of seismic instruments has steadily been increasing. The International Seismological Centre (ISC) currently lists 11,447 reporting stations that are distributed globally. However, fewer than 500 of these (4.2%) are located in the open ocean. Despite efforts by academic groups to distribute very high quality broadband instruments more evenly, a recent list of 909 stations belonging to the Federation of Digital Seismic Stations (FDSN) has less than 10% of its total in the oceans away from the coast (7 stations in the Indian Ocean, 19 in the Atlantic and 62 in the Pacific). Given that the oceans cover almost 2/3 of the Earth’s surface, this uneven sensor distribution causes problems for seismometry, not in the least for our efforts to image the Earth’s interior and understand the processes operating in the deep mantle. Whereas tomography has been very succesful in mapping shallow subduction anomalies (which are mostly located near dense networks on the coast), the fate of deeper sinking slabs is still controversial. For the mantle plumes that carry hot rock upwards, the situation is even worse, because almost all of them are located in the oceanic domain. Slabs and plumes are considered to be workhorses for heat transport in the mantle, but their exact role in the management of the Earth’s heat budget (as opposed to mantle-wide convection) is still open to many questions^1,2^.

Though ocean bottom seismographs (OBSs) have been used in efforts to compensate for the paucity of sensors near several oceanic hotspots^3–5^, deploying them in the abundance and for the number of years required to reach sufficient resolution well into the lower mantle is very costly. In this paper we show how a new technology for seismometry in the oceans can bring enhanced resolution in the oceans, and present a first application to the Galápagos hotspot. Instead of OBSs, which reside at their launching location for the duration of an experiment, we deploy Lagrangian drifters equipped with a hydrophone and software to discriminate seismic signals from other acoustic sources, named MERMAIDs (Mobile Earthquake Recording in Marine Areas by Independent Divers) by Simons *et al*.^6^ MERMAIDs allow for two-way communication of data and operating commands by satellite, and provide a promising and affordable complement to land and ocean bottom seismometers for P wave tomography^7,8^. Our experiment with nine MERMAIDs in the Galápagos region constitutes the first application of this new instrumentation in a network configuration. The primary goal of the experiment was to evaluate the quality of the data and the usefulness of MERMAID deployments for regional tomography. But the target itself is one of only about two dozen deep mantle plumes^9^, and therefore of significant scientific interest in itself.

The Galápagos Archipelago is located almost 200 km south of the Galápagos Spreading Center (GSC). The buoyancy flux of the proposed deep Galápagos plume is estimated from the swell^9^ to be 10^3^ kg/s, which conforms to a heat flux of about 0.04 TW. Though relatively modest, it provides a convenient test for the validity of the flux estimates based on swell topography. The basalts on the islands show a high ^3^He/^4^He anomaly, pointing to a primordial mantle source at depth, though differences exist between the Sr, Nd, Pb, and Hf isotopic ratios which are more indicative of a plume for basalts from GSC further north than those from central and northeast Galápagos^10^. There may exist a shallow conduit between the observed top of a plume beneath the islands, and the GSC, an idea also supported by numerical modeling^11–13^. A receiver function analysis by Hooft *et al*.^14^ showed a thinning of the transition zone, such as to be expected for a hot plume originating in the lower mantle. Montelli *et al*.^15^ were the first to image a P-wave anomaly extending from the Galápagos islands down to 1800 km depth, but large differences with later tomographic efforts exist^16–18^. The exact origin of the Galápagos heat source is therefore still uncertain. The absence of a He isotope anomaly on the nearby spreading ridges even led Sallarès *et al*.^19^ to conclude that the hotspot is caused by a compositional anomaly involving recycled crust, rather than a deep mantle plume.

The existence of nearby land stations to the N and E of the archipelago makes this region a good testbed, where even a small number of MERMAIDS to the S and W can provide dense coverage with seismic rays where none existed so far.

## The MERMAID Experiment

**Figure 1:**
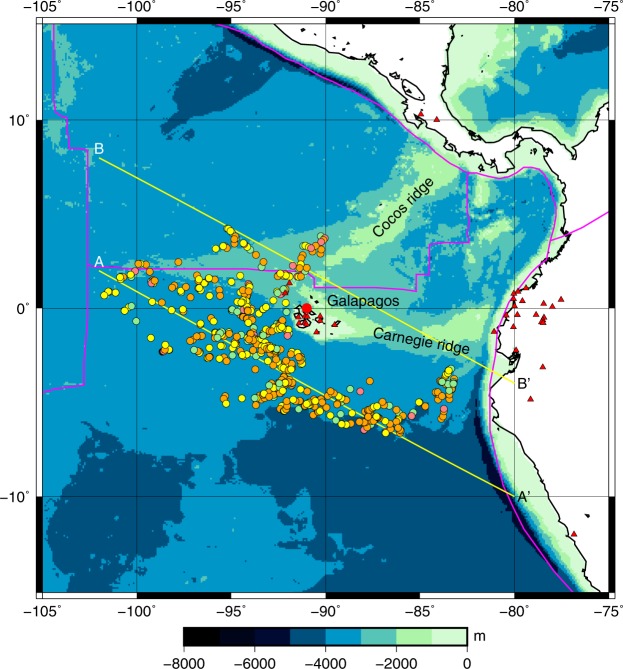
A bathymetric map of the Galápagos hotspot region. The locations of MERMAID floats at the times of the P wave arrivals is indicated by circles, where the colour indicates epicentral distance (red: Δ < 10°, orange: 10° < Δ < 30°, yellow 30° < Δ < 100°, green: Δ > 100°). Regional land seismometers used are indicated by red triangles. Plate boundaries are shown in magenta. Lines AA’ and BB’ denote the locations of the cross-sections shown in Fig. S3.

We launched the first MERMAID on May 7, 2014. The lifetime of these first-generation MERMAIDs, 21 months on average, was limited by the finite battery capacity, and thus determined by the variable activity of the CPU, the number of surfacings and the duration of data transmission after recording a signal in response to a trigger. If a P wave is converted to an acoustic wave at the ocean floor, the signal recorded by the hydrophone is essentially a seismogram, and P wave arrival times can be ‘picked’ to observe delays caused along the wave path. The last such seismogram was received on October 25, 2016 (Table 1). The instruments float passively with the abyssal current and this motion creates a natural array of closely spaced observations. Figure 1 shows the locations of the MERMAIDs at the time they recorded a seismogram. The MERMAIDs drift passively, normally at a depth of 1500 m.

**Table 1:** MERMAID instrument performance.

MERMAID nr	launch	last transmission	number of surfacings	data sent (kB)	months alive
10	2014-05-23	2015-09-11	101	2062	15
19	2014-06-04	2015-12-28	111	2047	19
20	2014-05-07	2016-10-13	138	2935	29
21	2014-05-07	2016-05-10	159	3263	24
22	2014-06-05	2016-10-13	154	4261	28
23	2014-06-05	2015-10-17	97	2007	16
24	2014-05-23	2016-06-04	196	4474	25
25	2014-05-08	2014-12-06	40	785	6
26	2014-05-08	2016-10-25	136	4146	28

Their trajectories reflect the turbulent nature of deep ocean currents (for an example see Fig. S1 in the Supplementary Materials). The median drift was 3.92 km/day (Fig. S2). Upon detecting a possible P wave signal, a MERMAID rises to the surface, usually in 95 minutes, and determines its position and the clock drift with GPS. On average, a MERMAID triggered every 5 days. Triggering is done using Sukhovich’s algorithm^20^ to analyse the hydrophone signal sampled at 40 sps and high-passed at 0.1 Hz; downsampling can be used to limit the cost and energy consumption of data transmission (the data for this paper were transmitted at 20 sps).

**Figure 2:**
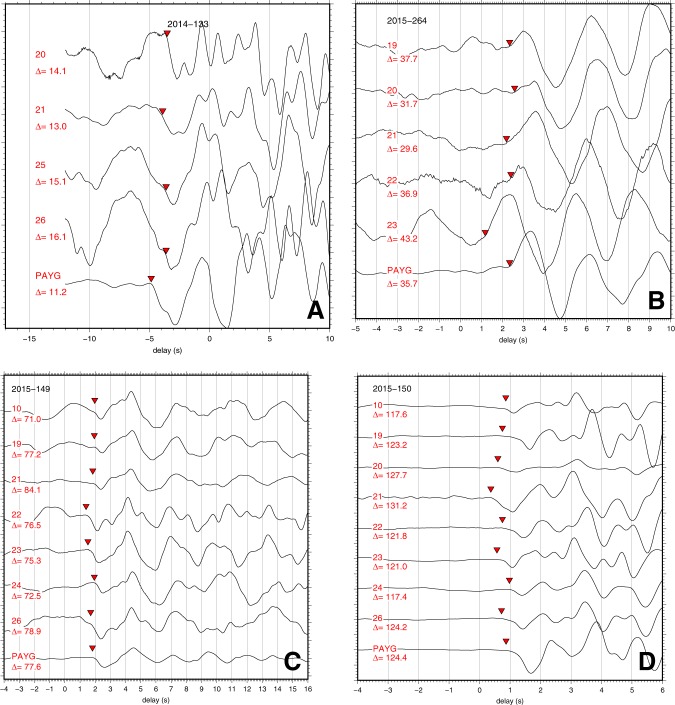
Examples of seismograms recorded by MERMAIDs for a range of epicentral distances. (**A**) May 13, 2014, 7.2 °N 82.3 °W, depth h = 10 km, Mw 6.5, (**B**) Sep 21, 2015, 31.7 °S, 71.4 °W, h = 35 km, Mw 6.6, (**C**) May 29, 2015, 56.6 °N 156.4 °W, h = 73 km, Mw 6.7, (**D**) PKP waves from May 30, 2015, 27.8 °N, 140.5 °E, h = 664 km, Mw 7.8. Delay is defined with respect to the predicted P wave arrival time for model AK135, inverted triangles indicate the picked onset. Distances Δ (in degrees) are listed with each recording. The records of GSN station PAYG, located in a borehole on the Galápagos Island of Santa Cruz, are listed for comparison.

Examples of seismograms are shown in Fig. 2. A total of 1329 signals were transmitted, of which 719 could be identified with an earthquake present in the NEIC catalogue. The remaining transmissions include many false triggers, though several appear to be small earthquakes not present in the catalogue. We had to reject about one in five seismograms because high noise prevented the picking of an accurate onset; we measured 580 arrival times, most of them with an accuracy of a few tenths of seconds. To avoid that some or all of the structural information contained in the MERMAID travel time delays is absorbed in corrections for earthquake origin time and hypocentral locations, we augment the MERMAID data with onsets that we picked for the same events from a handful of nearby stations of the national network of Ecuador (see triangles in Fig. 1), as well as from GSN stations with epicentral distances Δ < 90°. The total of 5,068 picks is referred to as the MERMAID (MM) data set. We embedded the regional MM data in a global inversion using a global data set of delays published by the International Seismological Centre (ISC – see the Methods section for details).

Plots of ray coverage (Fig. S3) shows that the gain in illumination with respect to the global data set, representative for data otherwise available for tomography, is especially significant in the upper mantle.

## Results

**Figure 3:**
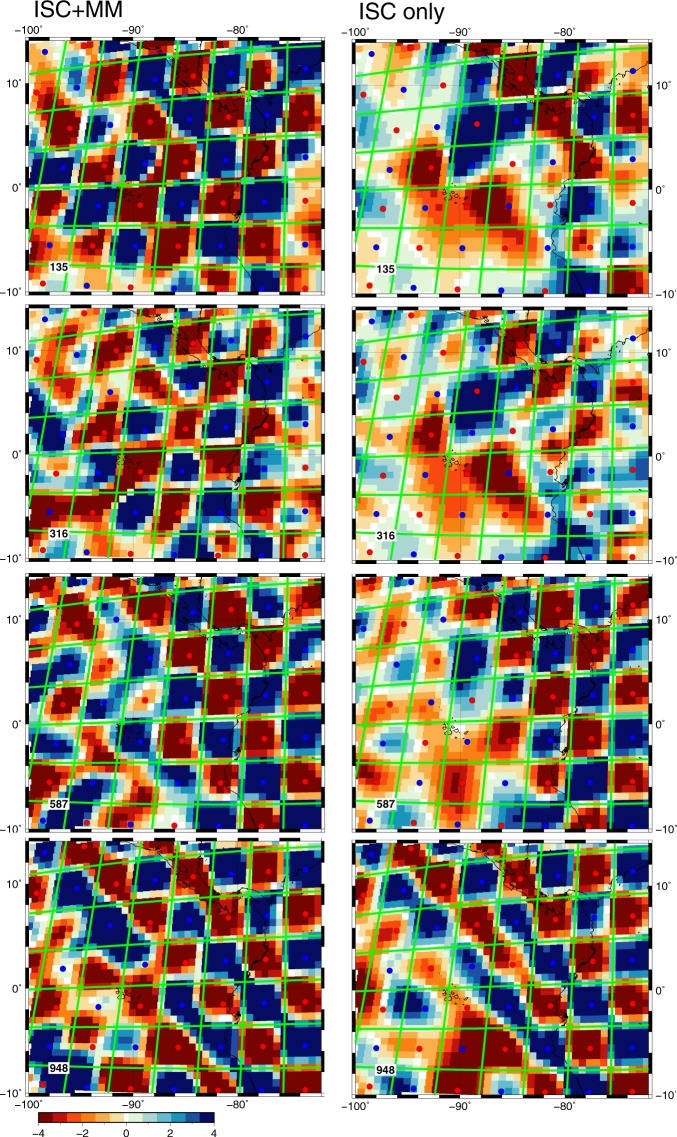
Resolution test for a checkerboard at depths of 135, 316, 587 and 948 km comparing (left) the improved resolution using ISC delays plus MERMAID data with (right) that for ISC delays only. Damping, smoothing and number of iterations was the same in both cases. Colour scale is in percent. The checkerboard boundaries (green lines) follow the cubed Earth parameterization and are 6 voxels wide. Circles indicate the (±2.5%) anomaly in the input model.

We carefully estimated the errors in the picked data and performed a separate data analysis for errors in the ISC data (see Methods section), such as to limit the range of possible solutions to those that fit the data to the level expected by the errors, but not less or more. Our preferred model fits the data to the estimated standard errors (reduced *χ*^2^ = 1.04). A checkerboard test of the increase in resolution is shown in Fig. 3. This test confirms that the increase obtained by the array of floating seismographs is significant in the upper mantle. Especially in the area NE of Galápagos, where P wavepaths from earthquakes in central America and the Caribbean cross each other, the gain in resolution offered by the MM data is major: whereas the sharpness of the checkerboard cells indicates a resolution close to that of individual voxels of about 70 km size, the test with just the ISC data shows correct resolution only near the coasts where land stations provide the information. Even in the transition zone at 587 km the improvement from float data is remarkable, and only in the lower mantle (948 km depth) do we see that the images converge. Because of the limited extent that one can reach with only 9 floats over the course of less than two years, the floats contribute rays that are dominantly close to vertical in the deeper part of the region. This complements raypaths towards land stations that have larger angles with the vertical, but their added value to the resolution is clearly less compared with that in the upper mantle. Yet there is at 948 km still a remarkable improvement in the SW corner, farthest away from the land stations.

**Figure 4:**
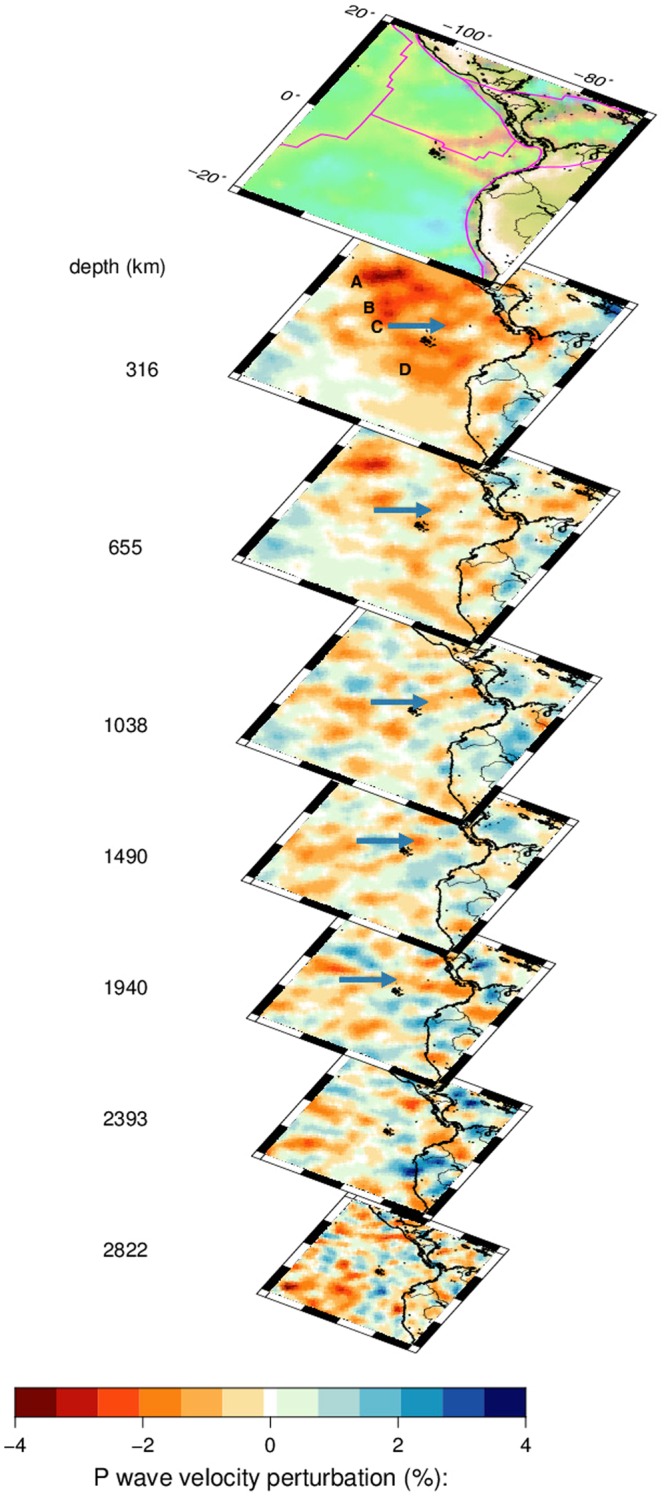
Three-dimensional image of the preferred tomographic solution for P-velocity anomalies. An arrow denotes a negative velocity anomaly that is continuous to a depth of about 1900 km, and that is imaged in the cross-sections of Fig. 5. The magenta lines denote plate boundaries. Upper mantle anomalies A-D are discussed in the text.

The three-dimensional image of our preferred solution for the mantle beneath Galápagos is shown in Fig. 4. In the upper mantle (depth 316 km) where the model is well resolved, we see a large region with negative velocity anomalies that vary in strength. The largest negative anomaly (labeled A) near 106 °W, 8 °N is under the East Pacific Rise and is an anomaly also imaged in recent efforts using waveform tomography^17,18^. Going in a southeastern direction from there, one perceives three elongated anomalies (B,C,D), one of which is located near the Galápagos. All three persist until the top of the lower mantle (depth 655 km). Anomaly C, just N of Galápagos then continues at 1038 and 1490 km depth and remains weakly visible until 1940 km depth.

**Figure 5:**
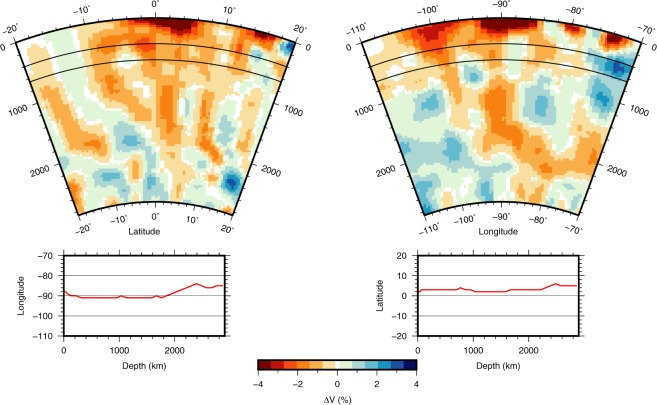
Tomographic result showing the P-velocity anomaly of the Galápagos plume in two perpendicular depth sections viewed from the East (left) or South (right). The cross sections are slightly warped with depth to track the maximum anomaly; deviations from 90 °W (left) and 0 °N (right) are plotted in the graphs below the plot. The transition zone between upper- and lower mantle is indicated by black lines at 410 and 660 km depth.

That this indeed is a continuous plume down to 1900 km depth can be seen from Fig. 5, in which we plotted cross-sections in a vertical plane that is slightly bent to follow the strongest anomaly, which we interpret as the center of the plume. A 200–300 km wide, almost vertical negative anomaly is located at the longitude of the Galápagos hotspot at about 91 °W. It tilts slightly from a depth of 1900 km. We interpret this as the deep plume feeding Galápagos. The continuation below 2000 km depth in the latitude section (Fig. 5, left) is visually misleading, since we have to warp the plane strongly to the East to follow this anomaly, whereas the longitude section 5, right) indicates a clear break. The resolution test for a 2000 km deep plume (Fig. S4) shows some leakage to greater depth, which reinforces our opinion that the plume does not fully extend to the core-mantle boundary.

## Discussion

We have shown that precise onset times of P and PKP waves from moderately strong events can be observed with floating seismographs. Even a limited number of floats can cover a large region, thanks to their continuous movement which serves to avoid duplications in raypaths and limits the redundancy of the data. The average longevity of the MERMAIDS used in this study was rather short at 21 months. This average includes one of the MERMAIDs that lived only six months. The cause of its sudden disappearance is not known. In one rare instance a PP wave triggered data transfer, indicating that we may have missed information by tuning the trigger algorithm to higher frequency P waves and/or by restricting data transfer to the first arrival only and by limiting the length of the transmitted seismograms. The main lessons we learned from this experiment are that significant and affordable new information can easily be obtained with floating seismographs in the oceans, that significant improvements in data yield can be made by prolonging the lifetime of the floats, and also that more data can be retrieved if – besides the automatic transmission following a trigger – data can be requested on command from a buffer. These improvements have since been implemented in a new MERMAID design that uses a spherical float with a three times larger battery capacity. With this increase in lifespan, we expect future MERMAID deployments to spread out over a significantly wider area, depending on the local ocean circulation pattern. This will increase resolution to even greater depths than obtained in this study.

The succesful picks of onset times came from earthquakes with magnitudes as low as 5 (Fig. S5). But the majority of teleseismic readings came from magnitudes higher than 6. Because of their high frequency content, deep quakes are easier to pick than shallow quakes. There does not seem to be a correlation between magnitude and depth of hypocentre. We conclude that the local weather conditions dominate in creating favourable signal-to-noise conditions. As can be seen in Fig. 2D, signal-to-noise in the MERMAIDs compares well with that in borehole station PAYG if the earthquake is strong and the microseismic noise low.

The ability to move with the ocean current sets the floats apart from OBSs. For seismic tomography, this advantage compensates for the lack of shear wave information, though best results can probably be obtained by combining the two types of instruments since their capabilities are so different. Compared to OBSs, the MERMAIDS are very easy to deploy. Both deployment and recovery can be done by one person from small ships, not necessarily scientific vessels. Recovery may not be cost effective in the open ocean as one day’s ship time is usually more expensive than the cost of the instrument itself. However, the increase in battery lifespan of the new version (5–6 years) makes it probable that they will sooner or later drift into coastal areas where retrieval with small ships is affordable. The ability to view the data in quasi-real time, to have clock corrections done by GPS at every surfacing and to be able to manage the data stream automatically is also a time- and cost-savings characteristic that sets the MERMAIDs apart from OBSs. On the other hand, OBSs are free from the cost of data transmission, which depends on the parameters set by users for event triggers and length of seismograms. Transmitting seismograms of length 200–250 s, the cost of sending data per Iridium satellites, using Rudics protocol, was about $ 100 per float per month in our experiment. Transmission costs also depend on the appetite of the user, who defines sampling rate and duration.

Despite the limitations in this first experiment, our tomographic results are of interest for geodynamics. We consider our error estimates reliable, both for the picked data and for the ISC delays, which limits the range of allowable damping parameters, and which therefore reduces the uncertainty in anomaly amplitudes to a useful level (see Methods section and Table 2).

**Table 2:** Plume temperature anomalies (°K) at depth 655 and 1400 km calculated for different solutions.

Solution	1400 km r = 50 km*	655 km r = 50 km	655 km r = 250 km	Damping
a	748	256	180	Preferred solution with *χ*^2^ = 1
b	470	185	105	Overdamped to *χ*^2^ = 1.2
c	784	345	166	Less smoothing, *χ*^2^ = 1
d	713	279	135	Less smooting, column scaling, *χ*^2^ = 1

*Radius of the circular region over which Δ*T* is averaged.

The anomaly spreads out in the upper mantle near the surface, and has a maximum slightly north of the equator where the Cocos and Carnegie Ridges meet. The depth resolution of our cubed Earth parameterization is not sufficient to obtain a detailed image of melt migration near the surface and towards the GSC north of the island chain from the observed shallow P and Pn waves, but the general pattern of low velocity near the surface supports the hypothesis of a deflection of plume material towards the GSC, which might explain the observation of Sr, Nd, Pb, Hf anomalies at the GSC, whereas the He has escaped at an earlier stage to cause an anomaly limited to the island basalts as suggested by Villagomez *et al*.^12^.

The high temperature of the Galápagos plume argues for an important role of the plumes in the Earth’s heat budget. The values listed in Table 2 give an idea of the variability of temperature estimates in a range of models that satisfy the data. If we adopt the temperature derivatives given by Nolet *et al*.^21^ we find that the large velocity deviation of −2.1% at 1400 km depth gives a Δ*T* of 913 °K. This, however, is only a point value in one voxel, and averaging over an area with a radius of 50 km centered at the minimum in P-velocity lowers it to 748 °K, or as low as 470 °K if we apply extra damping allowing for a less ideal, though probably still acceptable, data fit. The uncertainty in *dV*_*p*_/*dT* adds to extend the range of allowable temperature variations, but this midmantle estimate is certainly much higher than the moderate temperature anomaly observed by Hooft *et al*.^14^ of 130 ± 60 degrees in the transition zone. When averaged over a region of 500 km diameter, our temperature estimates at 655 km are in agreement with those found from the thinning of the transition zone, lending credence to our adopted *dV*_*p*_/*dT*. The mid-mantle temperature is also above the range of 75–214 °K inferred for the upper mantle from other geochemical and geophysical observations^22^. Since we expect the temperature gradient in the plume to be close to adiabatic, this observed increase in excess temperature with depth measures the subadiabatic gradient of the ambient mantle. Numerical modeling by Bunge^23^ indicates that a subadiabiatic gradient in the mid-mantle implies internal heating and a large contribution of core heating to the dynamics of the mantle, which is also in agreement with the viewpoint of a more important role of plumes than is often assumed^1,2^.

We tried an order-of-magnitude calculation for the flux into the transition zone, assuming an average rise of *v* = 5 cm/yr over a 200 km wide plume, a heat capacity *C*_*p*_ of 1250 J/kg/K, a density *ρ* of 4400 kg/m^3^ and Δ*T* = 250 °K. With this we obtain an estimate for heat flux into the upper mantle at 660 km of *πr*^2^*vC*_*p*_*ρ*Δ*T* = 8.6 × 10^18^ J/yr or 0.27 TW, which is a factor of 6–7 larger than that estimated from the swell. If we could generalize this factor to the estimate of 2.5 TW for all plumes, the total plume heat flux would be of the order of 15 TW. This matches the flux across the 660 km discontinuity that remains missing when one subtracts the (cold) flux contribution of the sinking slabs from the observed mantle heat flux^1^, and would imply that there is no other heat exchange between lower and upper mantle besides that carried by slabs and plumes. However, this is just a very rough calculation and to answer this question more definitely, we need to have more accurate flux estimates and for many more plumes. Sukhovich *et al*.^8^ calculated that a global fleet of 1000 MERMAIDs, operating for 5 years in the oceans, would be able to image plumes in mid-mantle with the resolution needed to estimate their widths and P-velocity anomaly with high precision.

Although an almost vertical continuation between 1900 km depth and the core-mantle boundary at 2890 km seems visible in the section along longitude in Fig. 5 (right), we must bend the plane by several hundred km to keep following the anomaly, and this is physically not likely to be the same plume anomaly. Rather, the extension visible to 70 °W at 2000 km seems to indicate a wide and shallow source region.

It may be a coincidence, but the depth of about 1900 km for the source of the upwelling matches the depth of the top of a mantle reservoir proposed by Kellogg *et al*.^24^ to be the source of enrichment in primordial ^3^He and ^4^Ar for deep mantle plumes. Our observations indicate that the Galápagos plume is ultimately fed by heat produced in such a reservoir, by high flux from the core, or – most likely – by both.

We conclude that Galápagos is fed by a 1900 km deep, 200–300 km wide, almost vertical plume beneath 0 °N, 91 °W with a large temperature anomaly, which is separate from a stronger upper mantle upwelling beneath the East Pacific Rise further west. We note that the observed width of the Galápagos plume is consistent with the size of upwellings needed to explain the distribution of isotope ratios in the uranium series in comparable hotspots^25^.

## Methods

### Errors in onset time

**Figure 6:**
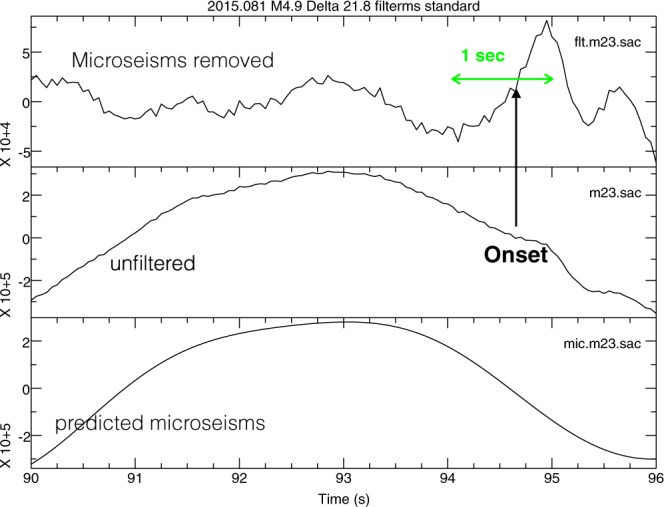
The result of filtering the seismograms. We subtract the estimated microseismic signal (bottom plot) from the observed seismogram (center), to obtain the filtered signal (top) used for picking the onset time.

Microseismic noise impedes the use of cross-correlation for accurate measurement of delays, except for a few very strong arrivals. We therefore resorted to the classical ‘picking’ of the onset of P and PKP wave arrivals. Since they follow a minimum-time path, the travel time for such onsets can be exactly computed with ray theory^21^. If the P wave has sufficient energy for frequencies above about 0.5 Hz (which is usually the case for hypocentres below crustal depths), the onset is clearly visible even if the microseismic noise is high. We correlate onsets ‘by eye’ between MERMAIDS and nearby land stations. For shallow events, the frequency bands of P and noise largely overlap, and even strong events with magnitudes >6.5 can easily be missed by the trigger or their onsets can be difficult to read. For these data we developed a filter to remove much of the microseismic noise. To avoid any pre-causal artefacts it does not use FFT, but fits instead a Fourier series of finite length to the time signal of *T* seconds:

1 $${s}_{m}(t)=\sum _{n=1}^{{n}_{{\max }}}\,[{a}_{n}\,\cos \,n{\omega }_{0}t+{b}_{n}\,\sin \,n{\omega }_{0}t],$$

where *ω*_0_ = *π*/*T* and *n*_*max*_ depends on the noise spectrum but is typically such that frequencies are limited to the 0–0.4 Hz band. We then subtract *s*_*m*_(*t*) from the observed seismogram *s*(*t*) before picking the arrival. An example is shown in Fig. 6. Even for shallow events this allows us often to pick the time of the onset with an uncertainty limited to one or two samples.

There are, however, indirect data errors that must also be taken into consideration. The first one is caused by errors in the float location. When the float records the signal it is deep beneath the surface and out of reach of GPS, so that we have to estimate its recording location from float drift measurements, after it surfaces. The associated location error is equivalent to a timing error in the onset – the steeper the incident ray, the smaller is this error. We measure the surface drift (median: 1.35 km/h) using two or more GPS fixes while the MERMAID transmits its data to the Iridium satellite network. This enables us to pinpoint the location of the float’s arrival at the surface very precisely. We measure the abyssal drift assuming a constant velocity between the current position and the previous surfacing, and thus determine the float’s position at cruising depth at the time of earthquake detection and recording. The correction for the total drift in the water column during ascent is typically less than 400 m. The equivalent time correction for a mantle P wave is less than 0.05 s, and the expected error even smaller. If the float did not rise immediately to the surface the error can be about three times as large^26^, but these cases are rare (most of the triggered signals that were stored without direct surfacing turned out to be false triggers). The depth of the float is accurately determined by the pressure sensor with an accuracy of a few meters and does not contribute to the over-all data error.

The tomographic inversion software includes a correction for crustal structure based on model CRUST2.0^27^ which averages over cells of 2° × 2° in longitude and latitude. This is much larger than the width of the Fresnel zone of a 1 Hz P wave at the ocean bottom, which varies with the bathymetry but is typically only a few km, and introduces an error in the length of the water column and thus in the crustal correction. By comparing the bathymetry of CRUST2.0 with that of ETOPO1^28^ on a 10 km grid where the ocean is deep enough to host a MERMAID we estimate that 78% of the bathymetry errors are less than 300 m, or equivalent time errors <0.15 s.

The variance in delays caused by location and bathymetry must be added to the variance ${\sigma }_{e}^{2}$ due to picking errors in the onset time. Combining location and bathymetry errors with onset reading errors that were determined subjectively, our data have been assigned errors in a range between 0.3 s for sharp onsets, and occasionally as high as 1.2 s if the onset is emergent and the noise level is high. The actual picking error may be larger in the case of noise wrongly picked as onset, but such errors are outliers that are removed if the residual delay after a first inversion exceeds 3*σ*_*e*_.

### Embedding in global tomography

Most of the picked P waves have part of their wave path outside the region of interest, and we must therefore prevent that distant anomalies elsewhere influence the imaging beneath the Galápagos. For this reason we embed the MM data in a global inversion of P and PKP delays reported by the ISC. We selected the events from the recently revised ‘EHB’ data set that minimizes mislocation bias^29^, at the time of our analysis available for the period 2000–2003. Events with less than 180° azimuth coverage, or fewer than 15 stations reporting were rejected. The ISC data set contains few raypaths crossing the upper part of the mantle under the Galápagos, hence the MERMAID data were crucial in providing the regional illumination shown in Fig. S3. The origin time and hypocentre for the ISC data were allowed to vary in the inversion. The data vector *d* is to a high approximation linearly related to the vector of model perturbations and source corrections *m*:

2 $$Am=d.$$

This system is scaled to unit prior variance for both data and model elements^21^. To determine the standard error in the ISC data, we adapt the method of Voronin *et al*.^30^, and organize the data in event clusters (rather than parallel ray paths). The tomographic submatrices (*A*_*c*_) for these clusters contain highly redundant information and if the data are projected on the eigenvectors of the matrix ${A}_{c}{A}_{c}^{T}$ they have a distribution characterized by the data standard error *σ*_*e*_ as well as the standard devation *σ*_*m*_ of the true delays, caused by the Earth’s heterogeneity. The variance of the datum projected on the *i*–th eigenvector with eigenvalue ${\lambda }_{i}^{2}$ is given by

3 $${\sigma }_{i}^{2}={\sigma }_{m}^{2}{\lambda }_{i}^{2}+{\sigma }_{e}^{2}.$$

**Figure 7:**
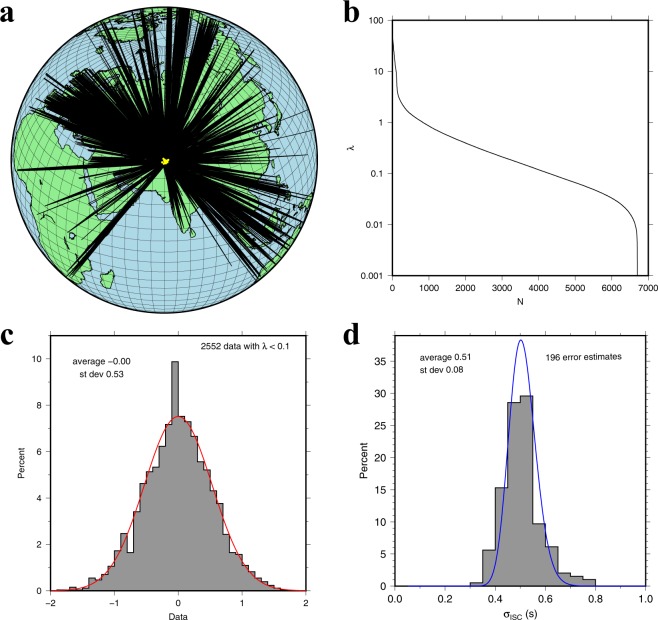
(**a**) An example of ISC delay standard error estimation over a cluster of closely spaced earthquakes using the method of Voronin *et al*.^30^. In this case we selected 38 events (yellow dots) and diagonalized the tomographic submatrix for their N = 6842 wavepaths (solid lines). (**b**) The distribution of the 6842 eigenvalues (logarithmic scale). The rapid fall-off indicates a large redundancy caused by overlapping raypaths. (**c**) the distribution of the projected data for the 2552 smallest eigenvalues (eigenvalue *λ* < 0.1). These eigenvalues are associated with directions in data space that are dominated by noise, allowing an estimate of the standard error in the data (0.53 s for this selection). The red curve shows the best fitting normal distribution, (**d**) averaging over the estimated errors from 196 such clusters we determined that the standard error in the ISC-EHB data was 0.51 s. The blue curve shows the best fitting lognormal distribution.

Because of the scaling to unit variance in the data and unit prior variance of the model parameters that we applied to (2), *σ*_*e*_ and *σ*_*m*_ are dimensionless and of order 1, and the distribution of projected data with ${\lambda }_{i}^{2}\ll 1$ is thus dominated by ${\sigma }_{e}^{2}$, enabling an objective estimate of the standard error in the data. Using this on 196 large clusters we determined an average standard error of 0.51 ± 0.08 s for the ISC P-delays (Fig. 7), which is much less than an early estimate of 1.4 s by Morelli and Dziewonski^31^ for the ISC P-delays. It is at the low end of the 0.4–0.9 s range found by Gudmunsson *et al*.^32^ for errors at teleseismic distances only.

This error estimation requires clusters of data that are large, and may not be representative for delays from very isolated events, which we therefore decided not to use. After these selections, we used 1,539,758 P delays. We also selected 237,166 PKP delays. The PKP data set is too small to estimate their errors reliably from event clusters and we assigned a standard error of 1 s to PKP delays, which includes delays acquired in the core, which is not included in the tomographic model. After a first inversion run with very low damping, we rejected 11.3% of data that could not be fitted within 3*σ*_*e*_. This reduced the total data set to 1,579,503 data (9.1% of the MM data were rejected). Delay times are defined with respect to the predicted times for model AK135^33^. To parameterize the P velocity anomalies in the mantle we used a cubed sphere with 37 layers and 6 mantle sections of 128 × 128 voxels for a total of 3,637,248 voxels^34^. The average horizontal voxel size at the surface is 72 km. Most layers are 90 km thick, though we accommodate discontinuities by halving some layers. The linear system is augmented by regularization constraints and solved using a parallel version of LSQR^21^.

### Regularization

The large number of voxels in the cubed Earth calls for a regularization dominated by smoothing. Knowledge of the errors allowed us to calibrate the regularization such that the misfit *χ*^2^ is exactly the misfit imposed by normally distributed errors, thus considerably reducing the uncertainty in the amplitudes of anomalies that often hampers tomographic images (see next section). Most importantly, this allowed us to get temperature estimates for the plume at depth that still have a large uncertainty, but that are more precise than can be inferred from more arbitrarily damped tomographic solutions.

In addition to a norm damping, we bias the the solution towards smoothly varying models by minimizing absolute spatial derivatives. We precondition the system (2) by substituting *m* = *Sy* and first solving for a minimum norm solution *y*, where *S* is one step in a relaxation scheme that solves ${\nabla }^{2}y=0$ (it averages voxel *y*_*i*_ over its neighbours – this is essentially a discretized version of a scheme originally proposed by VanDecar and Snieder^35^, extended to three dimensions). Since we consider our estimates of standard errors to be reliable, we solve for a data fit that has a reduced *χ*^2^ close to 1. In summary, the system we solve is:

4 $$(\begin{array}{c}A\\ {\varepsilon }_{n}I\\ {\varepsilon }_{s}D\end{array})\,Sy=(\begin{array}{c}d\\ 0\\ 0\end{array}),$$

followed by

5 $$m=Sy.$$

*I* is the unit matrix and *D* differences model parameters but not the source corrections^21^. *ε*_*n*_ and *ε*_*s*_ are weights for norm damping and smoothing, respectively. Except for two alternative inversions (see below) we kept the ratio *ε*_*n*_/*ε*_*s*_ = 0.05. After 2500 iterations of LSQR the data misfit agreed to 4 figures with its final value, but it required 8500 iterations to converge to the optimal fit of the regularization constraints. The resulting model for the mantle under the Galápagos has a ${\chi }_{red}^{2}$ of 1.04 (i.e. the average misfit to the data is $\sqrt{1.04}$ or 1.02 standard errors).

### Resolution analysis

The checkerboard resolution test shown in Fig. 3 was done by combining the voxels of the cubed Earth model into cells of 6 × 6 voxels horizontally and 5 voxels vertically, leaving the top crustal layer unperturbed. Checkerboard cells have an average horizontal dimension of 408 km in the upper mantle. The top checkerboard has a thickness of 338 km, followed by two layers of 315 and 405 km, respectively, after which the cells are 450 km thick. These dimensions define the dominant wavelength in the resolution test, but the fact that the cells have sharp corners implies that there is still significant energy at much shorter wavelengths. We assumed constant anomalies of ±2.5% in each cell, computed synthetic data and added normally distributed errors with the same standard deviation as estimated for the real data. We then inverted these data using the same damping parameters as was used for the preferred model.

For the tests in Fig. S4 we replaced the checkerboard with a plume that has a maximum anomaly of 2.5% which decreases from the center as a Gaussian function with a (1/e−) diameter of 200 km.

Finally, to test the sensitivity of the solution to the damping, we performed three more inversions of the real data. Figure S6a shows again the cross-section along the equator of the preferred model. If we underfit the data somewhat to obtain a still acceptable ${\chi }_{red}^{2}=1.20$, we find the model shown in Fig. S6b. Keeping the optimal data fit (${\chi }_{red}^{2}\approx 1.00$), but doubling the weight of the damping in the *ε*_*n*_/*ε*_*s*_ ratio gives the model shown in Fig. S6c. Finally, if we weight the columns such as to reduce the effect of ray density on the minimization, by weighting columns inversely proportional to their norm (using a waterlevel to avoid excessive error propagation), we get the model shown in Fig. S6d. All models show a plume beneath Galápagos going down to about 1900 km. Below that depth the algorithm that follows the plume has warped the cross-section more forcefully in models (b–d) than in model (a). This explains the difference near the core-mantle boundary, but in no case is there a vertically continuous plume all the way down to that interface, since one has to move up as far as 10 °N to find a low-velocity connection (and this even from 1400 km depth in the case of model b).

## Supplementary information

Supplementary figures
