# Supplementary material for: Imaging the Galápagos mantle plume with an unconventional application of floating seismometers
Source: Sci Rep. 2019 Feb 4;9:1326. doi: 10.1038/s41598-018-36835-w (PMC6362208; doi:10.1038/s41598-018-36835-w)
Supplement: Supplementary file 1 — Supplementary figures [file 41598_2018_36835_MOESM1_ESM.pdf]

Imaging the Galápagos mantle plume with an unconventional application of floating seismometers.

Guust Nolet[1,2,3], Yann Hello[1], Suzan van der Lee[4], Sebastien Bonnieux[1,+], Mario C. Ruiz[5], Nelson A. Pazmino[6], Anne Deschamps[1], Marc M. Regnier[1], Yvonne Font[1], Yongshun J. Chen[3] and Frederik J. Simons[2].

1. Université de la Côte d'Azur/CNRS/OCA/IRD, Géoazur, Sophia Antipolis, 06560, France.
2. Department of Geosciences, Princeton University, Princeton, NJ 08540, USA.
3. School of Ocean Science and Engineering, SUSTech, 518055 Shenzhen, China.
4. Department of Earth and Planetary Sciences, Northwestern University, Evanston, IL60208, USA.
5. Instituto Geofísico, Escuela Politécnica Nacional, 2759 Quito, Ecuador.
6. INOCAR, 5940 Guayaquil, Ecuador.

\+ Now at: Université de la Côte d'Azur/CNRS, Laboratoire i3s, 06560 Sophia Antipolis, France.

Supplementary Materials

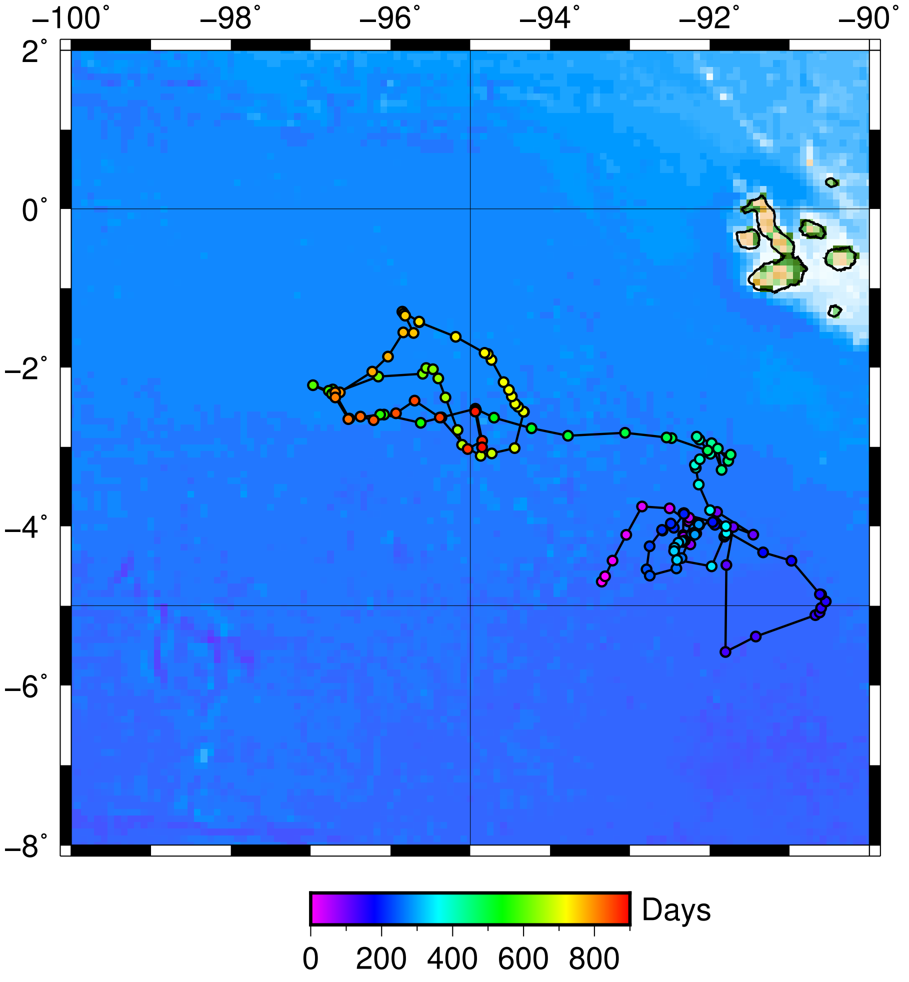

Figure S1: The path travelled by MERMAID 26 illustrates the turbulent, quite unpredictable nature of the abyssal drift in this area. Every circle denotes a surfacing, its colour indicates the number of days since launch. The total length of this trajectory is 3313 km, the duration 891 days.

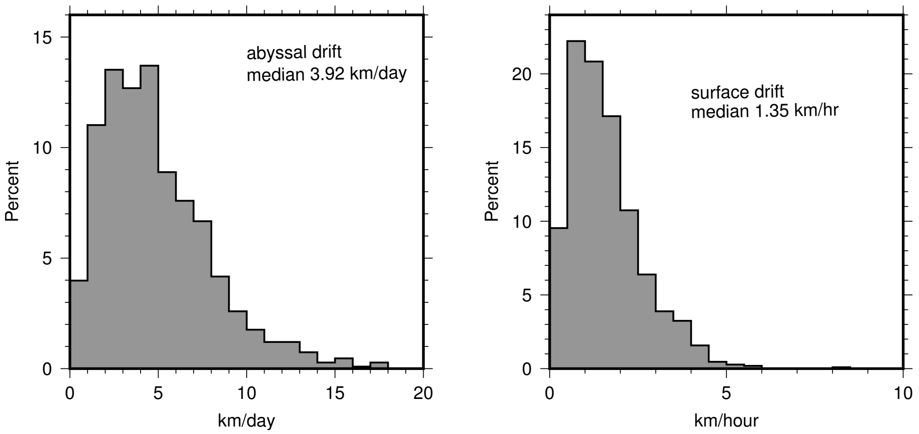

Figure S2: Drift statistics near the Galápagos as measured from the first 1086 MERMAID surfacings. Left: abyssal drift rate measured by differencing two consecutive surfacings, assuming a linear trajectory. Right: surface drift rate measured by differencing two or more GPS fixes, made while the MERMAID remained at the surface for data transmission.

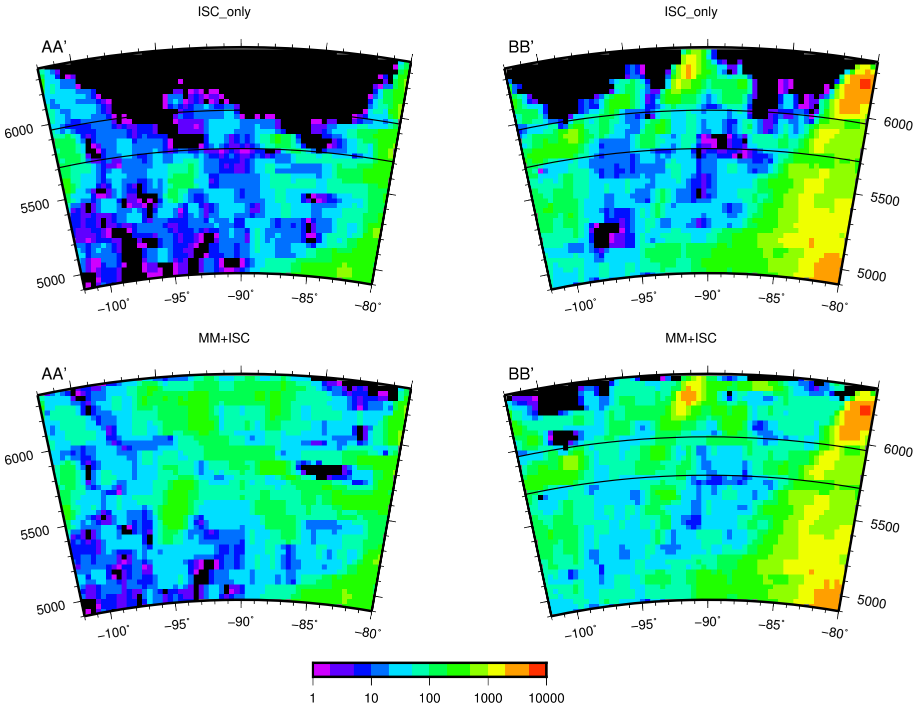

Figure S3: Improvement of the tomographic illumination of the mantle provided by MERMAID data. Spatial sampling of rays is represented by the L1 column norms of the tomographic matrix in two cross sections for ISC data only (top), and for the data including the MERMAID arrival times (bottom), along lines AA' and BB' shown in Figure 1. Warm colours indicate a larger number of rays sampling the region. The top row shows ray density provided by ISC data only, the bottom row is for the combined ISC+MM data set.

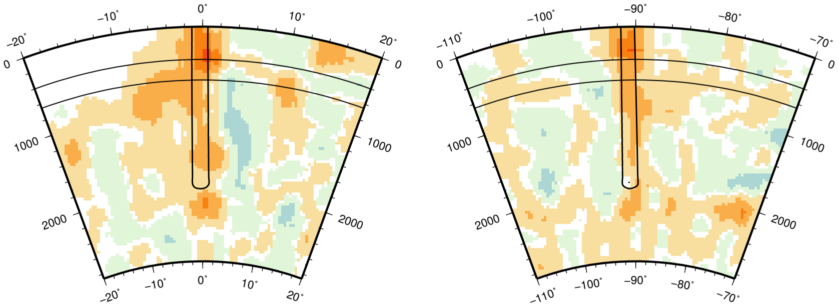

Figure S4: The resolution of the data (including simulated noise), tested for a synthetic, vertical, plume model shown at 0N,91W (not warped) with a maximum negative anomaly of 2.5%. The input plume used to generate synthetic data over the same raypaths as the real data extends to 2000 km depth and has an anomaly that decreases with a Gaussian profile. Its diameter (defined by a decrease to 1/e of the maximum) is 200 km. The colour scale shows the image produced with data calculated for this model after adding Gaussian noise with the same error as estimated for the real data. The input plume contour line is at a level of −1%. The colour scale is as in Figure 5.

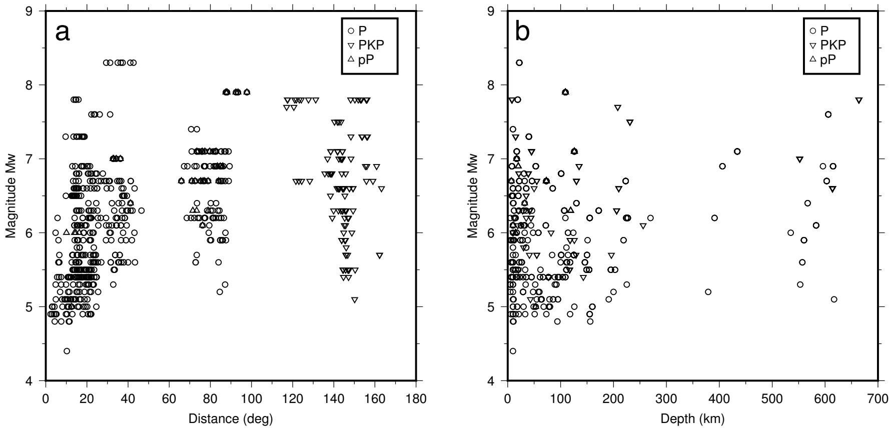

Figure S5: (a) Observed magnitudes as a function of epicentral distance, (b) and as a function of hypocentre depth. The absence of a clear correlation in both indicates that the weather (absence of storms) is probably the major factor that decides observability.

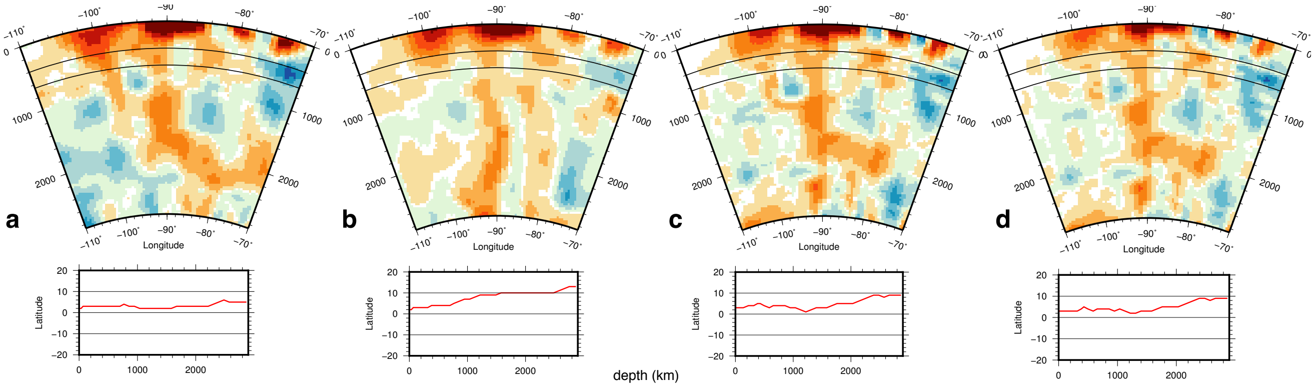

Figure S6: Comparison of longitude sections for the models listed in Table 2. All models shown have an acceptable data fit, but differ in the choice of regularization. The warping, shown in the bottom row of figures, tracks the maximum anomaly, and differs between models. The colour scale is as in Figure 5.
